# Potential Benefits of Coffee Consumption on Improving Biomarkers of Oxidative Stress and Inflammation in Healthy Individuals and Those at Increased Risk of Cardiovascular Disease

**DOI:** 10.3390/molecules28186440

**Published:** 2023-09-05

**Authors:** Phiwayinkosi V. Dludla, Ilenia Cirilli, Fabio Marcheggiani, Sonia Silvestri, Patrick Orlando, Ndivhuwo Muvhulawa, Marakiya T. Moetlediwa, Bongani B. Nkambule, Sithandiwe E. Mazibuko-Mbeje, Nokulunga Hlengwa, Sidney Hanser, Duduzile Ndwandwe, Jeanine L. Marnewick, Albertus K. Basson, Luca Tiano

**Affiliations:** 1 Cochrane South Africa, South African Medical Research Council, Cape Town 7505, South Africa; mn.muvhulawa@gmail.com (N.M.); duduzile.ndwandwe@mrc.ac.za (D.N.); 2 Department of Biochemistry and Microbiology, University of Zululand, Richards Bay 3886, South Africa; hlengwan@unizulu.ac.za (N.H.); bassona@unizulu.ac.za (A.K.B.); 3 Department of Life and Environmental Sciences, Polytechnic University of Marche, 60131 Ancona, Italy; ilenia.cirilli@unicam.it (I.C.); f.marcheggiani@univpm.it (F.M.); s.silvestri@univpm.it (S.S.); p.orlando@univpm.it (P.O.); l.tiano@staff.univpm.it (L.T.); 4 Department of Biochemistry, North-West University, Mafikeng Campus, Mmabatho 2735, South Africa; mtdmoetlediwa@gmail.com (M.T.M.); sithandiwe.mazibukombeje@nwu.ac.za (S.E.M.-M.); 5 School of Laboratory Medicine and Medical Sciences, University of KwaZulu-Natal, Durban 4000, South Africa; 6 Department of Physiology and Environmental Health, University of Limpopo, Polokwane 0727, South Africa; sidney.hanser@ul.ac.za; 7 Applied Microbial and Health Biotechnology Institute, Cape Peninsula University of Technology, Bellville 7535, South Africa; marnewickj@cput.ac.za

**Keywords:** metabolic disease, diabetes, cardiovascular disease, oxidative stress, inflammation, coffee consumption

## Abstract

Cardiovascular diseases (CVDs) are considered the predominant cause of death globally. An abnormal increase in biomarkers of oxidative stress and inflammation are consistently linked with the development and even progression of metabolic diseases, including enhanced CVD risk. Coffee is considered one of the most consumed beverages in the world, while reviewed evidence regarding its capacity to modulate biomarkers of oxidative stress and inflammation remains limited. The current study made use of prominent electronic databases, including PubMed, Google Scholar, and Scopus to retrieve information from randomized controlled trials reporting on any association between coffee consumption and modulation of biomarkers of oxidative stress and inflammation in healthy individuals or those at increased risk of developing CVD. In fact, summarized evidence indicates that coffee consumption, mainly due to its abundant antioxidant properties, can reduce biomarkers of oxidative stress and inflammation, which can be essential in alleviating the CVD risk in healthy individuals. However, more evidence suggests that regular/prolonged use or long term (>4 weeks) consumption of coffee appeared to be more beneficial in comparison with short-term intake (<4 weeks). These positive effects are also observed in individuals already presenting with increased CVD risk, although such evidence is very limited. The current analysis of data highlights the importance of understanding how coffee consumption can be beneficial in strengthening intracellular antioxidants to alleviate pathological features of oxidative stress and inflammation to reduce CVD risk within the general population. Also covered within the review is essential information on the metabolism and bioavailability profile of coffee, especially caffeine as one of its major bioactive compounds.

## 1. Introduction

Coffee is a highly commercialized food product, which is considered one of the most consumed beverages globally [1,2]. As with any plant extracts, coffee contains a complex mixture of bioactive compounds [3], but the specific composition of these compounds varies depending on the type of coffee, as well as its roasting and processing method [4]. Chlorogenic acids are the most abundant form of phenolic compounds found in coffee, potentially driving its enhanced therapeutic effects against oxidative stress [5]. Research has explored the potential bioactivity of the phenolic metabolites of chlorogenic acids [6,7]; however, these findings have not been fully established. On the contrary, the effects of coffee on promoting plasma antioxidant capacity and antioxidant enzymes, leading to the inhibition of damage to proteins and lipids, have been reported [8,9]. It is also acknowledged that coffee consumption, depending on the type, as well as the dose and duration of the intervention, can also cause overproduction of reactive oxygen species (ROS) [10]. Enhanced production of ROS, together with reduced intracellular antioxidants may give rise to oxidative stress, the major consequence implicated in the progression of metabolic complications [11].

**Figure 1:**
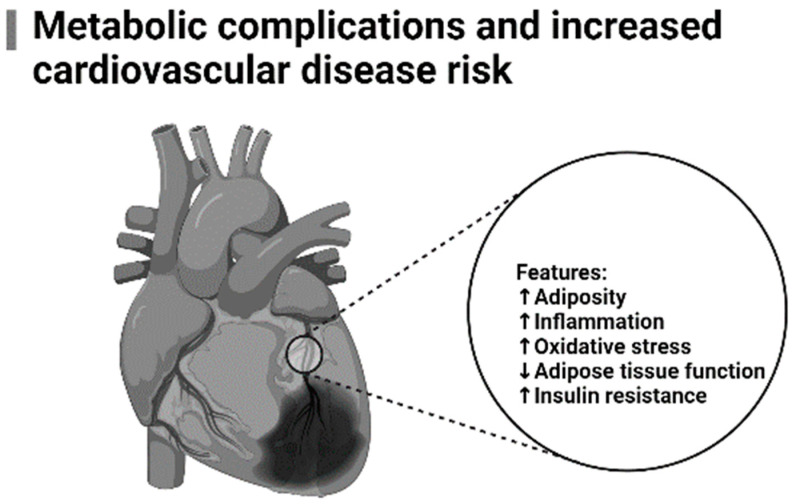
A general overview of pathophysiological mechanisms associated with increased cardiovascular disease risk. This is with especial focus in individuals with metabolic syndrome, displaying excess adiposity (adipose tissue dysfunction), that is concurrent to state of inflammation, insulin resistance and oxidative stress. The arrows (within the image) facing up show increased/acceleration of pathological features (including adiposity, inflammation, oxidative stress, and insulin resistance), while the arrow facing down indicates reduced adipose tissue function, which are linked with the development of cardiovascular disease, especially in conditions of metabolic disease.

Actually, both oxidative stress and inflammation are continuously investigated for their involvement in the pathogenesis of cardiovascular diseases [12,13]. Oxidative stress can trigger the activation of diverse transcription factors that prompt the initiation inflammation [14]. Increased biomarkers of oxidative stress and those that favor a pro-inflammatory state, including malonaldehyde (MDA), together with interleukin (IL)-6, tumor necrosis factor alpha (TNF-α), and high sensitivity C-reactive protein (hs-CRP) have been correlated with increased cardiovascular disease (CVD) risk [12,13,15], and this is more pronounced in conditions of metabolic disease [16,17]. Although commonly used medications can be beneficial in correcting underlying complications of the metabolic disease, including reducing blood glucose levels or blocking cholesterol synthesis [18,19], prolonged use of some of these therapies may aggravate the detrimental effects of oxidative stress and inflammation [20,21]. Some of the pathophysiological consequences of the metabolic disease driving increased CVD risk are summarized in Figure 1.

In fact, there is an interest in determining whether antioxidant therapies can be used in combination with common drugs to alleviate CVD-related complications in individuals with metabolic diseases [22,23,24,25]. Although drawbacks that may be associated with the dose or even duration of the intervention is acknowledged [8,9,10], accumulative evidence suggests that coffee consumption can be beneficial against CVD-related complications [26,27,28]. Others even report an inverse correlation between coffee consumption and all-cause and CVD mortality [29]. This extends to the alleviation of complications related to hypertension, type 2 diabetes (T2D), and blood pressure, which may lead to improvements of the general human health [30,31,32,33]. Nevertheless, information directly reporting on the modulation of biomarkers of oxidative stress and inflammation in response to coffee consumption during disease pathogenesis remains scanty. This highlights the importance of the current review, evaluating the potential use of coffee as a preventative or protective strategy against the development of CVDs, which may be through effective modulation of biomarkers of oxidative stress and inflammation in healthy individuals or those at increased risk of developing CVDs. The current review also covers essential information on the metabolism and bioavailability profiles of coffee, which is necessary to determine its potential health benefits.

## 2. Methodology for Study Inclusion

Briefly, a systematic search was performed by accessing data from major electronic databases including PubMed, Google Scholar, and Scopus, to identify relevant studies. The search strategy was compiled using the following keywords or Medical Subject Headings (MeSHs); “coffee”, “oxidative stress”, and “inflammation” including most relevant synonyms as well as related keywords of the search topic. The literature search was conducted from inception until June 2023, while a manual search was performed to identify additional relevant studies. Because of their quality of evidence [34], the systematic search focused on randomized controlled trials reporting on any correlation between coffee consumption and the modulation of markers of oxidative stress and inflammation. The search as well as data analysis was independently performed by two authors to identify and scrutinize relevant studies. Tables were created to summarize relevant data items, including author details, the country where the study was conducted, dose and duration of coffee, as well as the main findings for each study, focusing on the modulation of markers of oxidative stress and inflammation.

## 3. Metabolism and Bioavailability Profile of Coffee, Especially Caffeine as One of Its Major Bioactive Compounds

Coffee contains ripe seeds of Coffea arabica Linn., which are part of the Rubiaceae family. Approximately 75% of the world’s production of coffee is provided by Coffea arabica, while an estimated 25% and less are contributed through other species like Coffea canephora and Coffea liberica [35]. Countries where coffee is considered native include Brazil, Ethiopia, India, Indonesia, Mexico, Nepal Guatemala, Sri Lanka, and Vietnam [35]. Analytical techniques, including high-resolution mass spectrometry, mono- and bidimensional nuclear magnetic resonance are routinely used to determine the chemical composition or in quantifying the phenolics present in coffee fractions [36]. The chemical composition of coffee beans is very intricate; however, carbohydrates are considered the predominant chemical constituent found in coffee [37]. Other chemical constituents found in coffee beans include proteins, fats, tannins, caffeine, minerals, and many other bioactive compounds in small amounts. However, carbohydrates account for approximately 60% of the total weight of raw coffee beans. Diverse factors influence the chemical composition of raw coffee beans, especially the roasting process, which is likely to influence the unique flavors and colors of various coffee beans [8]. Reviewed information suggests that the roasting process is critical at determining the chemical changes in the main constituents of green coffee beans, including carbohydrates, chlorogenic acids, trigonelline, proteins, and free amino acids, together with the formation of aliphatic acids, aroma components, and melanoidins [38]. Many studies have performed the pharmacokinetics profile of caffeine, which is relatively well-understood [39,40,41].

Coffee encompasses a variety of bioactive compounds that affect the human body such as caffeic acid, chlorogenic acids, trigonelline, diterpenes, and melanoidins [42]. As one of the major bioactive compounds present in coffee [43], most of the pharmacokinetic studies have explored the use of caffeine in the form of solutions, capsules, and tablets to understand its metabolism and absorption [44,45]. Other studies have also investigated the bioavailability of coffee’s phenolic and chlorogenic acids due to the complex metabolic pathways involved in humans [46,47]. However, it is known that these chlorogenic acids can be transformed into phenolic acids such as caffeic, ferulic, and isoferulic moieties, which can further be converted into colonic metabolites like dihydrocaffeic and dihydroferulic acids. Extensive conjugation through intestinal and liver metabolism can lead to the identification of various metabolites from a cup of coffee, including aglycone, sulfate, glucuronide, and methyl [46,47]. While lactones, diterpenes, cafestol, kahweol, niacin, and vitamin B3 are present in coffee, caffeine is the most bioactive component of most coffee products [48].

**Figure 2:**
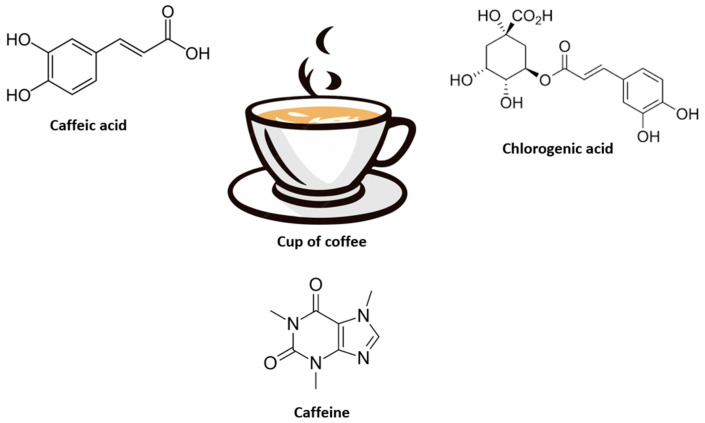
Caffeic acid, chlorogenic acid, and caffeine remain some of the predominant bioactive compounds found in coffee. Notably, chlorogenic acids can be transformed into phenolic acids such as caffeic, ferulic, and isoferulic moieties, which can further be converted into colonic metabolites like dihydrocaffeic and dihydroferulic acids. While extensive conjugation through intestinal and liver metabolism can lead to the identification of various metabolites from a cup of coffee, including aglycone, sulfate, glucuronide, and methyl. Caffeine is highly absorbed after ingestion and remains actively involved in the bioactivity of coffee.

Caffeine is extensively metabolized in liver cells to form various compounds such as dimethylxanthines, monomethylxanthines, dimethyluric acids, monomethyluric acids, and uracil derivatives [39]. The major enzymes involved in caffeine metabolism are phase I cytochrome P450 (CYP) enzymes, particularly CYP1A2, which accounts for approximately 13% of total P450 enzyme content in the human liver [49]. Also, CYP1A2 isoform is responsible for almost 90% of caffeine metabolism [50,51]. Other enzymatic pathways involved in caffeine metabolism include CYP1A1, CYP2E1, CYP2A6, monooxygenase, and N-acetyltransferase activities [52]. Paraxanthine is the primary caffeine metabolite in plasma, while methylated xanthines and methyluric acids are the primary metabolites excreted in urine [53]. Upon ingestion, caffeine can rapidly or almost completely be absorbed into the bloodstream, with 20% of the absorption occurring in the stomach and the remaining 80% in the small intestine [54,55]. It can also be absorbed quickly through the oral mucosa [56], the route that is independent of digestive system pathways [56,57]. In human subjects, the bioavailability of caffeine from orally administered coffee has been demonstrated to be 3.5 times higher than that from coffee enema [40]. A coffee enema involves injecting room temperature coffee into the rectum, as it is also considered a type of colon cleanse applied as alternative medicine. Other studies showed that the time to reach peak plasma concentration after oral doses of 72 to 375 mg of caffeine varies between 15 and 60 minutes, even 120 min in cases of oral administration, depending on various factors, including the type of food matrix, volume, solid or liquid, capsule, gum, and individual physiology [58]. Caffeine absorption from soda and chocolate is slightly delayed relative to coffee [58], while absorption from a chewing gum format is faster [56] than in coffee or capsules [57]. After oral consumption of 70–500 mg of caffeine, peak plasma concentration varies between 1.1 and 17.3 µg/mL [53,59,60]. However, actual plasma concentration may be higher in daily dietary intake, while thorough bioavailability profile of coffee has been previously reviewed [61]. Some of the major bioactive compounds, and their absorption profiles are described in Figure 2.

## 4. Characteristic Features of Included Clinical Studies

The systematic search produced 18 relevant studies reporting on the link between coffee consumption and modulation of biomarkers of oxidative stress and inflammation in healthy subjects, as well as individuals at increased risk of CVD (Table 1). Reported literature was mainly from countries in Europe and South America, while a few studies were from the United States, Thailand, and Taiwan, of which are all countries being acknowledged to be one of the leading consumers of coffee globally [62]. Summarized literature mainly included data on the modulation of oxidative stress and inflammation biomarkers from healthy individuals, while a few studies involved participants with obesity, hypercholesterolemia and T2D, which are some of the risk factors for CVD [63]. Covered information entails different types of coffee, which include instant, filtered, and roast coffee (Table 1). While the intervention period ranged from as short as 90 min, and up to, or predominantly 4–8 weeks.

### 4.1. Evidence on the Effects of Coffee Consumption on Biomarkers of Oxidative Stress

Table 1 provides an overview of evidence reporting on the potential benefits of coffee consumption in improving biomarkers of oxidative stress in healthy individuals and those at increased CVD risk. Evidence emerged as early as 2005 showing that healthy individuals receiving two cups of filtered coffee (300 mL/day) for 3 weeks displayed increased plasma homocysteine levels, while also not affecting lipid peroxidation [64]. These findings suggesting that coffee consumption does not alleviate CVD risk in this study population, since both plasma homocysteine levels lipid peroxidation are increased in people with cardiovascular complications [77,78,79]. Such limitations have also been confirmed by a few others showing that consumption of coffee does not influence lipid peroxidation, while also leading to increased biomarkers of liver function in healthy individuals [70,72,75]. However, a study conducted in 2012 in healthy individuals receiving instant roast coffee (482 mL/day) for 4 weeks showed that although lipid peroxidation was unaffected, these individuals presented with increased antioxidant status, including increased plasma levels of catalase (CAT), superoxide dismutase (SOD), and glutathione peroxidase (GPx) [65,68], perhaps indicating that the type of coffee or even prolonged intake of coffee can have a more pronounced effect in modulating biomarkers of oxidative stress in healthy individuals. To verify this hypothesis, other findings indicate that consumption of roasted coffee or coffee rich chlorogenic acids for 4 to 8 weeks can improve plasma antioxidant capacity, while decreasing biomarkers of lipid peroxidation (MDA levels) in some individuals [71,73,74]. Others went as far as showing that coffee consumption for 8 weeks can slow the skin aging process and improve skin health by neutralizing radical scavenging activity and inhibiting of tyrosinase activity in healthy individuals [76]. However, interesting evidence showed that regular consumption of roast coffee (approximately 500 mL/day) for 4 to 8 weeks can reduce the body weights, including improving the antioxidant status and lowering blood pressure in obese or hypercholesteremic subjects [67,73]. Enhancement of antioxidant capacity, including increasing in intracellular antioxidants like glutathione (GSH), SOD, and GPx appear to be the presumed mechanism by which consumption of coffee protects against formation of endogenous oxidative DNA-damage [66,67,68], as has also been reviewed by others [32].

### 4.2. Evidence on the Effects of Coffee Consumption on Biomarkers of Inflammation

Table 2 provides an overview of evidence reporting on the potential benefits of coffee consumption in improving biomarkers of inflammation in healthy individuals, including those at increased risk of developing CVDs. Apparently, not many clinical studies have reported on the modulation of inflammatory biomarkers in response to coffee consumption (Table 2). In fact, available information supports the beneficial effects of coffee consumption on improving subclinical inflammation and high-density lipoprotein (HDL) cholesterol, while significant changes were also observed for serum concentrations of interleukin-18, 8-isoprostane, and adiponectin in healthy individuals receiving four cups of filtered coffee for 4–8 weeks [80]. Those receiving coffee for a much shorter time (≤4 weeks) seem to not benefit as these individuals did not show improvements in appetite or levels of plasma glucose, or serum concentrations of insulin and inflammation [81,82]. Further indicating that significant changes or improvements inflammation response are likely dependent on the prolonged intake of coffee, as has been reported elsewhere [83]. However, individuals who are obese or type 2 diabetic receiving a diet rich in cereal fiber, free of red meat, and high in coffee for 8 weeks showed improved cardiac vagal function and oxidative glucose utilization. This diet even promoted a reduction in body weight and heart rate, which occurred independent of fluctuations in inflammatory status [84]. Furthermore, cyclists receiving high chlorogenic acid coffee for 2 weeks showed improved total mood disturbance scores, which also occurred independent of fluctuations in oxidative or inflammatory status [85]. This further affirms that long term consumption of coffee is more beneficial compared with short-term intake, even in physically active individuals.

## 5. Conclusions

**Figure 3:**
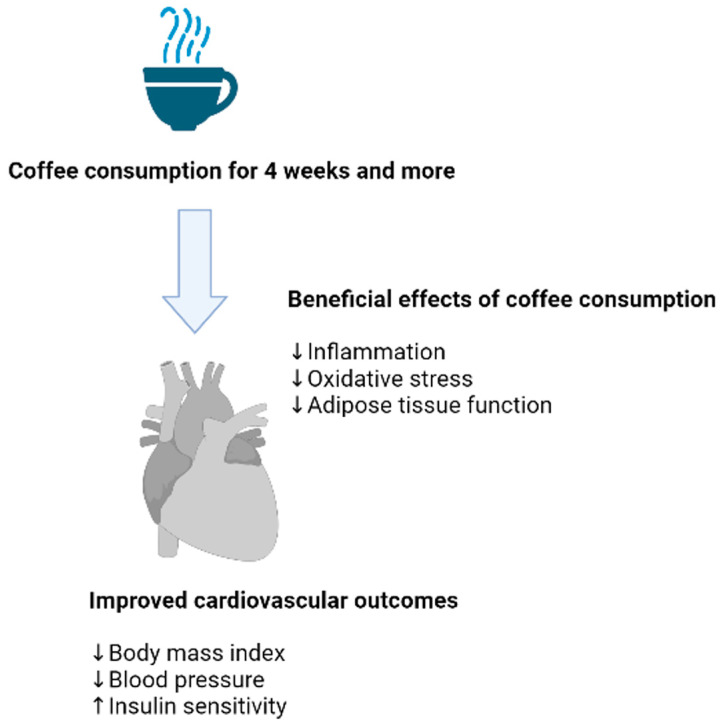
Regular consumption of coffee, especially for periods longer than 4 weeks is associated with improved biomarkers of oxidative stress and inflammation in participants who are overweight or obese. With reduction in blood pressure and body mass index, including improved insulin sensitivity, some of the markers related to alleviation of cardiovascular disease risk. Briefly, enhanced intracellular antioxidant response is the major mechanism to promote cardiovascular health within the human body, although such evidence has been covered through preclinical studies. The arrows (within the figure) facing down indicates reduced body mass index and blood pressure, while an arrow facing up designates enhanced insulin sensitivity.

Currently, CVDs remain the leading cause of death globally [86], while the pathological features of oxidative stress and inflammation are predominantly implicated in the development and acceleration of CVDs [87,88]. Plant-derived bioactive compounds and nutritional supplements are increasingly explored for their capacity to neutralize the detrimental effects of oxidative stress and inflammation to potentially reduce the increased risk of CVD [89,90,91]. As one of the leading increasingly consumed beverages [92], research has indicated that tea or its active ingredients can improve biomarkers of oxidative stress and inflammation to potentially reduce CVD risk in various disease conditions [93,94,95,96]. Data from the current review similarly indicates that coffee consumption, potentially due to its abundant antioxidants, is associated with the improved biomarkers of oxidative stress and inflammation in healthy individuals (Table 1 and Table 2). With some studies even showing that coffee consumption can reduce biomarkers of oxidative stress and inflammation to alleviate CVD risk in individuals with metabolic complications (Figure 3). This is consistent with preclinical evidence [42,97,98] on the proposed mechanisms by which coffee alleviates the toxic effects of oxidative stress and inflammation within diverse tissues to enhance its potential benefits. Enhancement of intracellular antioxidants via activation of nuclear factor erythroid 2-related factor-2 (Nrf2) is likely the leading mechanism that explains enhanced therapeutic effects of coffee or its bioactive compounds [99,100,101], as previously reviewed with other active ingredient found in other natural beverages [102,103]. This opens avenues for more research to further decipher how coffee or its active ingredients modulate intracellular mechanisms that are related to impaired cardiovascular systems, including energy metabolism, lipid metabolism, glucose uptake, and others. However, very limited evidence is available on the modulation of biomarkers of oxidative stress or inflammation with already increased CVD risk. The current analysis of data also does not underestimate the importance of understanding how coffee consumption can be essential in strengthening intracellular antioxidants to alleviate oxidative stress and inflammation to protect against opportunistic complications related to increased CVD risk in healthy subjects. Nonetheless, beyond improving lipid profiles, the current data suggest that prolonged use of coffee can be instrumental in improving biomarkers of oxidative stress and inflammation in the general population. Future research should be focused on enhancing our understanding on the influence of different doses of coffee (especially comparing men and women), concomitant to determining how other factors like the addition of milk contributes to its potential therapeutic benefits. In fact, other means to deliver the benefits coffee can be through enriching food sources like chewing gum with its bioactive compounds like caffeine to potentially improve human health.

## Figures and Tables

**Table 1:** Evidence on the potential benefits of coffee consumption in improving biomarkers of oxidative stress in healthy individuals and at increased cardiovascular disease risk.

**References**	**Country**	**Study Population**	**Intervention**	**Main Findings**
Mursu et al., 2005 [64]	Finland	Healthy nonsmoking men (n = 45), with age range of 20–26 years	Received two cups of filtered coffee (300 mL/day) for 3 weeks	Improved plasma homocysteine levels, although did not affect markers of lipid peroxidation
Moura-Nunes et al., 2009 [65]	Brazil	Healthy individuals (n = 10), with age range of 22–57 years	Received instant coffee (200 mL) for 90 min	Improved antioxidant capacity, correlating with uric acid and α-tocopherol
Mišík et al., 2010 [66]	Austria	Individuals (n = 38), with age range of 19–36 years	Received filtered coffee (800 mL/day) for 5 days	Protected against formation of endogenous oxidative DNA-damage. But did not affect levels of malondialdehyde (MDA), glutathione (GSH), intracellular reactive oxygen species (ROS) levels and the activities of superoxide dismutase (SOD) and glutathione peroxidase (GPx) in lymphocytes
Kotyczka et al., 2011 [67]	Germany	Obese individuals (n = 30), age not disclosed	Received roast coffee (500 mL/day) for 4 weeks	Reduced body weights, consistent with improving the antioxidant status of erythrocytes, including enhancing levels of SOD, GPx. Whereas tocopherol and GSH concentrations were also increased
Corrêa et al., 2012 [68]	Brazil	Healthy individuals (n = 20), with age range of 20–65 years	Received medium light roast and medium roast coffee (482 mL/day) for 4 weeks	Both coffees increased antioxidant status, including plasma levels of catalase (CAT), SOD, and GPx. But did not affect lipid peroxidation
Teekachunhatean et al., 2012 [69]	Thailand	Healthy individuals (n = 11), with age range of 13–30 years	Received ready to drink coffee (approximately 500 mL) daily for 11 days	Serum antioxidant status was improved, but did not directly affect GSH or MDA levels
Agudelo-Ochoa et al., 2016 [70]	Colombia	Healthy individuals (n = 75), with age range of 20–60 years	Received filtered coffee (400 mL/day), with either high or low chlorogenic acids for 8 weeks	Acute effect on the plasma antioxidant capacity, although did not have an effect on blood lipids or vascular function
Katada et al., 2018 [71]	Japan	Healthy individuals (n = 15), with age range of 20–60 years	Received rich in chlorogenic acids (185 mL) for 4 weeks	Effective in reducing chlorogenic acids-induced fat oxidation, while enhancing the antioxidant status
Shaposhnikov et al., 2018 [72]	Norway	Healthy individuals (n = 160), with age range of 35–65 years	Received five cups of filtered coffee for 8 weeks	Increased serum creatinine and the liver enzyme γ-glutamyl transaminase, while not affecting markers of oxidation of DNA and lipid
Martínez-López et al., 2019 [73]	Spain	Hypercholesterolemic individuals (n = 52), with age range of 18–45 years	Received 6 g/day of soluble green/roasted (35:65) coffee for 8 weeks	Improved plasma antioxidant capacity, while decreasing markers of lipid peroxidation (MDA), while reducing systolic and diastolic blood pressure, including heart rate and body weight
Lara-Guzmán et al., 2020 [74]	Colombia	Healthy individuals (n = 74), with age range of 20–60 years	Received two types of coffee that provided 787 mg chlorogenic acids/day (Coffee A) and 407 mg chlorogenic acids/day (Coffee B) for 8 weeks	Both coffees decreased urine oxylipins, while coffee A showed a stronger effect in reducing prostaglandins and prostaglandin metabolites. However, neither of the two coffees reduced the levels of oxidized low-density lipoprotein (oxLDL)
Martini et al., 2021 [75]	Italy	Healthy individuals (n = 21), with age range of 22–24 years	Received one/three cup of espresso coffee/day, and one cup of espresso coffee plus two cocoa-based products containing coffee for 4 weeks	No significant modulation of DNA and lipid damage markers was recorded, although DNA strand breaks and some markers of lipid peroxidation were modulated
Tseng et al., 2022 [76]	Taiwan	Healthy individuals (n = 40), with age range of 35–55 years	Received a coffee pulp drink (50 mL/day) for 8 weeks	Slowed the skin aging process and improved skin health. The radical scavenging activity was enhanced through inhibition of tyrosinase activity

**Table 2:** Clinical evidence of the impact of coffee consumption in biomarkers of inflammation.

**References**	**Country**	**Study Population**	**Intervention**	**Main Findings**
Kempf et al., 2010 [80]	Germany	Healthy individuals (n = 47), younger than the age of 65 years	Received 4 cups of filtered coffee/d and in the third month 8 cups of filtered coffee/d (150 mL/cup) for 4 weeks	Showed beneficial effects on subclinical inflammation and high-density lipoprotein (HDL) cholesterol, although not affecting glucose metabolism. Significant changes were also observed for serum concentrations of interleukin-18, 8-isoprostane, and adiponectin
Gavrieli et al., 2011 [81]	Greece	Healthy individuals (n = 16), with age range of 21–39 years	Received caffeinated coffee (3 mg caffeine/kg body weight) for 180 min before consuming meal *ad libitum*	Did not affect appetite-related ratings, the appetite plasma hormonal responses as well as the plasma glucose, serum insulin, and plasma and serum inflammatory marker responses. However, serum cortisol; cortisol concentrations were significantly higher following the caffeinated coffee intervention
Corrêa et al., 2013 [82]	Brazil	Healthy individuals (n = 20), with age range of 20–65 years	Received medium light roast and medium roast coffee (482 mL/day) for 4 weeks	Did not affect plasma total cholesterol, low-density lipoprotein-cholesterol, and soluble vascular cell adhesion molecule-1 concentrations. No changes were observed for lipoprotein, total homocysteine, glycemic biomarkers, and blood pressure
Ziegler et al., 2015 [84]	Germany	Obese and type 2 diabetic individuals (n = 28), with age range of 18–69 years	Received a diet high in cereal fiber, free of red meat, and high in coffee for 8 weeks	Improved cardiac vagal function and oxidative glucose utilization. Moreover, there was a reduction in body weight and heart rate with the heart rate variability being promoted. However, there was no effect on insulin sensitivity and inflammatory status
Nieman et al., 2018 [85]	United states	Cyclists (n = 15), with age range of 19–51 years	Received high chlorogenic acid coffee for 2 weeks	Improved total mood disturbance scores but did not affect blood inflammatory biomarker interleukin (IL)-6 and oxidative stress biomarker hydroxyoctade cadienoic acids

## Data Availability

Data related to search strategy, study selection, and extraction items will be made available upon request after the manuscript is published.

## Abbreviations

CAT: catalase; CVD: cardiovascular disease; GSH: glutathione; GPx: glutathione peroxidase; HDL: high density lipoprotein; MDA: malonaldehyde; HDL: high-density lipoprotein; hs-CRP: high sensitivity C reactive protein; IL: interleukin; Nrf2: nuclear factor erythroid 2-related factor-2; SOD: superoxide dismutase; T2D: type 2 diabetes; TNF-α: tumor necrosis factor alpha; ROS: reactive oxygen species.
